# Rationally Designed Ruthenium Complexes for Breast Cancer Therapy

**DOI:** 10.3390/molecules25020265

**Published:** 2020-01-09

**Authors:** Golara Golbaghi, Annie Castonguay

**Affiliations:** Organometallic Chemistry Laboratory for the Design of Catalysts and Therapeutics, INRS-Centre Armand-Frappier Santé Biotechnologie, Université du Québec, Laval, QC H7V 1B7, Canada; golara.golbaghi@iaf.inrs.ca

**Keywords:** ruthenium, breast cancer, multitargeted therapy, metallodrug, hormone positive breast cancer, triple negative breast cancer (TNBC), enzyme inhibition

## Abstract

Since the discovery of the anticancer potential of ruthenium-based complexes, several species were reported as promising candidates for the treatment of breast cancer, which accounts for the greatest number of new cases in women every year worldwide. Among these ruthenium complexes, species containing bioactive ligand(s) have attracted increasing attention due to their potential multitargeting properties, leading to anticancer drug candidates with a broader range of cellular targets/modes of action. This review of the literature aims at providing an overview of the rationally designed ruthenium-based complexes that have been reported to date for which ligands were carefully selected for the treatment of hormone receptor positive breast cancers (estrogen receptor (ER+) or progesterone receptor (PR+)). In addition, this brief survey highlights some of the most successful examples of ruthenium complexes reported for the treatment of triple negative breast cancer (TNBC), a highly aggressive type of cancer, regardless of if their ligands are known to have the ability to achieve a specific biological function.

## 1. Introduction

Cancer is a major public health issue worldwide [1,2]. More specifically, breast cancer is the most common cause of cancer death in women in developing countries and the second most common cause of cancer death in developed countries [3]. Although death rates for breast cancer dropped by 40% from 1989 to 2016 [1], specific types of breast cancer are still incurable [4]. Estrogen and progesterone receptors play a crucial role in the development of the most common breast cancer subtypes, and their expression is very highly predictive of their response to endocrine therapy. Different subtypes of breast cancer include estrogen receptor alpha (ERα), overexpressed in approximately 70% of invasive breast cancers, and progesterone receptor (PR), overexpressed in over two-thirds of estrogen receptor positive (ER+) breast cancers [5,6]. Another common biomarker in breast cancer is the epidermal growth factor 2 (HER2), overexpressed in approximately 20% of breast cancers [4]. Cancer patients who test positive for that protein can benefit from HER2-targeted therapy [7]. Finally, triple-negative breast cancer (TNBC), which represents 10–20% of all breast carcinomas [8], is characterized by the absence of ER, PR, and HER2 biomarkers [8,9]. Because of the ineffectiveness of endocrine therapy or therapies targeted to HER2 for TNBC, this type of cancer requires the development of different treatment approaches [8,10]. It is noteworthy that approximately 10–30% of patients with breast cancer, regardless of their hormone receptor status, develop metastases to the lymph nodes or/and distant organs, making the design of an efficient treatment for this heterogeneous type of breast cancer a challenging task [11,12]. Despite the associated short- and long-term risks, chemotherapy frequently remains an essential treatment for breast cancer, more particularly for late stage, metastatic, or triple-negative breast cancer [4]. Although patients with ER+ and/or PR+ breast cancer can benefit from endocrine therapy [4], alternative types of treatment including chemotherapy are often envisaged due to the side effects [13,14,15] and the high risk of post-therapy recurrence [16,17]. Besides, it was also reported that the combination of chemotherapy and endocrine therapy can significantly increase the survival rate of patients diagnosed with ER+ breast cancer [17].

Because of the wide range of coordination numbers and geometries, accessible redox states, and the thermodynamic/kinetic properties and nature of the coordinating ligands, inorganic compounds can exploit the unique properties of metal ions for the design of new anticancer drugs [18,19]. A well-known chemotherapeutic agent, cisplatin, is one of the most commonly used drugs to treat malignant breast cancers, either as a single agent or in combination with other drugs [20,21,22]. Although the success of cisplatin and its derivatives in breast cancer treatment is undeniable, these compounds usually display a range of severe side effects due to their lack of selectivity for cancerous over normal tissues [23]. The poor selectivity of cisplatin can be explained by its primary mode of action, which includes its interference with transcription and/or DNA replication mechanisms, not only limited to cancer cells but also to normal cells [23,24,25]. It has been reported that breast cancer cells can develop resistance to platinum-based drugs through different pathways, making it a major clinical obstacle to the development of successful treatments [26,27]. As an alternative to platinum-based chemotherapeutic agents, most efforts were devoted to the design of compounds based on ruthenium, as many were reported to display fewer side effects due to their different modes of action [28,29]. In many cases, ruthenium complexes were found to display a high cytotoxicity against platinum-resistant cancer cell lines, making them promising candidates for further investigation [30,31]. Also importantly, ruthenium species have demonstrated some promising activities in different types of breast cancer, opening the door to the design of novel metal-based chemotherapeutic agents. It is worth mentioning that several ruthenium complexes such as RAPTA-C [32], NAMI-A, and KP1019 [33] have successfully entered preclinical or clinical trials for the treatment of different cancers. Some ruthenium species also have the potential to act as photosensitizers (PS) in photodynamic therapy (PDT), which relies on the combination of a PS, light, and molecular oxygen. Upon light-activation, the excited state of the PS interacts with the ground state of molecular oxygen (^3^O_2_) to generate reactive oxygen species and notably singlet oxygen (^1^O_2_), which can interact with a wide range of biomolecules [34]. TLD-1433 is the first ruthenium(II)-based PS for PDT to enter a human clinical trial for the treatment of non-muscle invasive bladder cancer [35].

In recent years, the development of agents with enhanced anticancer properties via the coordination of biologically active molecules to metals, more specifically ruthenium, has attracted increasing attention [36,37,38]. Because ruthenium complexes are widely studied for their ability to induce cancer cell death, the introduction of biologically active ligands in their structure can be a promising approach for the development of drug candidates with a broader range of anticancer activities compared to ruthenium complexes or ligands alone [39,40]. This approach can potentially result in the creation of multitargeting drug candidates that could limit the emergence of cancer cell resistance mechanisms by leaving biological systems unable to compensate for the simultaneous action of two or more drugs [41]. The promising potential of this class of compounds was demonstrated by several rationally designed ruthenium complexes bearing biologically active ligands for the treatment of hormone receptor positive and hormone receptor negative breast cancers. For the former group of breast cancers, anticancer drugs including P450 inhibitors or steroid receptor-targeting molecules were included within the structure of the complexes whereas for the latter group, due to the lack of expression of hormone receptors, anticancer drugs with other modes of action (such as nonsteroidal anti-inflammatory drugs) were used as ligands. It is noteworthy that no ruthenium complex was specifically reported for the treatment of HER2+ breast cancer, so that reports of ruthenium complexes for hormone receptor negative breast cancer therapy are limited to TNBC therapy.

In this review, an overview of the rationally designed ruthenium-based complexes that were reported to date for breast cancer therapy is presented, with a special emphasis on species that include ligands that were carefully selected for the treatment of hormone receptor positive cancers, either estrogen receptor positive and/or progesterone receptor positive. In addition, this brief survey highlights some of the most successful examples of ruthenium complexes reported for the treatment of triple negative breast cancer, a highly aggressive type of cancer, regardless of if their ligands are known to have the ability to achieve a specific biological function. The information presented in this review is summarized in Table S1 (Supplementary Materials).

## 2. Ruthenium Complexes for the Treatment of Hormone Receptor Positive Breast Cancer

Anticancer drugs that are known to deprive cancer cells of the hormones they need for their growth, such as estrogen and progesterone, are promising ligand candidates for the design of multitargeting ruthenium complexes for the treatment of HR+ breast cancer. P450 enzyme inhibitors and steroid hormone receptor targeting moieties are examples of anticancer agents that are discussed in this section. Some successful examples of ruthenium complexes bearing other bioactive ligands such as nonsteroidal anti-inflammatory drugs (NSAIDs), epidermal growth factor receptor (EGFR) inhibitor, and glutathione S-transferase (GST) inhibitor are also presented.

### 2.1. Ruthenium Complexes Bearing P450 Enzyme Inhibitors 

**Figure 1:**
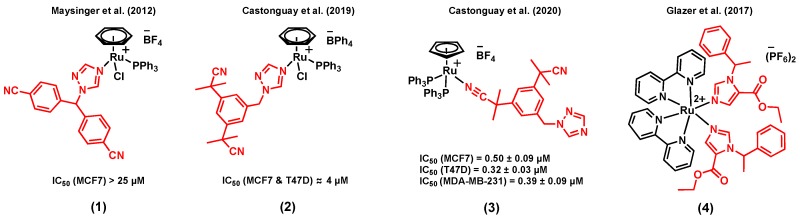
Ruthenium complexes bearing P450 enzyme inhibitors.

The combination of ruthenium with a P450 enzyme inhibitor in a single agent could potentially be beneficial for hormone receptor positive breast cancer therapy. P450 enzymes catalyze reactions that are involved in the biosynthesis and the metabolism of various important molecules [42,43]. For instance, the P450 enzyme aromatase (CYP19A1) plays a crucial role in steroid synthesis and thus in the growth of ER+ breast cancer [44]. Notably, it catalyzes the conversion of androgens to estrogens, a process known to provide the primary source of estrogens in postmenopausal women (for whom the production of estrogens is no longer governed by their ovaries) [45]. The mode of action of third-generation aromatase inhibitors is believed to take place via the N-interaction of their triazole ring with the iron of the enzyme’s cofactor, thus preventing the catalytic activity of the enzyme [46]. Furthermore, the inhibition of aromatase in breast tissues can also sensitize cells to chemotherapeutic agents [47,48]. Maysinger et al. (2012) reported the preliminary in vitro anticancer potential assessment of a series of ruthenium(II) arene complexes of the aromatase inhibitor letrozole in breast cancer cells, a clinically used third-generation aromatase inhibitor [49]. Among the ruthenium arene complexes reported in this study, complex (1) (Figure 1) showed the most promising cytotoxicity in the ER+ breast cancer cell line MCF7. Notably, the cytotoxicity of this compound was found to be significantly higher than that of the control complex [Ru(*η*^6^-C_6_H_6_)Cl_2_(PPh_3_)] (with no aromatase inhibitor), suggesting a contribution of the letrozole ligand on the activity of the compound [49]. However, the aromatase enzyme inhibitory potential of these complexes was overlooked. More recently, Castonguay et al. (2019) reported a series of ruthenium(II) arene complexes bearing a slightly different third-generation aromatase inhibitor, namely anastrozole [40]. In this study, the stability, in vitro cytotoxicity, in vitro aromatase inhibitory activity, and the in vivo toxicity of the complexes on the development of zebrafish embryos were investigated. Cationic complexes with a more lipophilic counterion (BPh_4_ vs BF_4_) showed a higher in vitro cytotoxicity, which could potentially be associated with greater levels of ruthenium cellular uptake, as measured by ICP-MS. Among all the synthesized species, the highest in vitro cytotoxicity was observed for complex (2) (Figure 1), which also displayed a high stability in cell growth media. An IC_50_ value of 4 µM was noted in both MCF7 and T47D breast cell lines, an activity significantly higher than that of the clinically-relevant drug cisplatin (IC_50_ > 150 µM, T47D; 37.0 ± 2.4 µM, MCF7). More importantly, the aromatase inhibitory activity of (2) was studied theoretically (by performing a docking simulation) and experimentally (using the tritiated water assay), which both showed a possible enzyme inhibitory activity for this compound, despite the involvement of the nitrogen atom of its triazole ring in the ruthenium coordination sphere. Also interestingly, no apparent in vivo toxicity (at 12.5 µM) was observed for this complex on the development of zebrafish embryos, which has become a prominent model for drug discovery and toxicity assessment [50]. Notably, more than 50% of zebrafish embryos treated with cisplatin under the same conditions could not hatch after 96 h, a clear indication of the toxicity of this chemotherapeutic agent [40]. Importantly, Castonguay et al. (2020) recently developed a novel ruthenium(II) cyclopentadiene (Cp) complex of anastrozole (3) (Figure 1) with a high in vitro cytotoxicity not only in ER+ breast cancer cells (IC_50_ = 0.50 ± 0.09 µM, MCF7; 0.32 ± 0.03 µM, T47D) but also in a TNBC cell line, MDA-MB-231 (IC_50_ = 0.39 ± 0.09 µM) [51]. Although this species was also cytotoxic in a non-cancerous breast cell line, MCF-12A (IC_50_ = 0.58 ± 0.02 µM), no apparent in vivo toxicity was observed on the development of zebrafish embryos at the tested concentrations. It is worth mentioning that both experimental and theoretical studies suggested that the interaction between this species and the aromatase enzyme is not likely to occur, most probably because of the bulkiness of the PPh_3_ moieties, preventing the compound from reaching the active site of the enzyme. Overall, the significant cytotoxicity of (3) against cancer cells, combined with its low toxicity on the development of zebrafish embryos, makes it an interesting candidate for further investigations. Some other P450 enzymes, such as CYP1B1, are also known to play a role in cancer initiation, progression, and drug resistance [52,53]. For instance, Glazer et al. (2017) reported an interesting study involving ruthenium(II) complexes bearing the P450 enzyme inhibitor etomidate, (4) (Figure 1), with dual enzyme inhibitory and DNA damaging activities upon light activation [54]. Although the drug candidate was not specifically designed nor tested for its activity in breast cancer, we reasoned that mentioning that study could be of interest to the reader as etomidate can also inhibit the activity of the aromatase enzyme [55,56,57]. Despite the interesting dual activity observed for this complex, its in vitro cytotoxicity was not studied [54].

### 2.2. Ruthenium Complexes Bearing Steroid Hormone Receptor Targeting Moieties 

Steroid hormones play a major role in regulating the expression of specific gene networks, and their biological effects on target tissues is mediated by specific receptors [58]. Several reports have shown that targeting hormone receptors in breast cancers can prevent their interaction with hormones and, as a result, block their function and lead to cancer cell death [59]. Thus, linking steroid hormone receptor-targeting moieties to metal-based drug candidates appears to be a promising avenue for the design of therapeutic agents [60].

**Figure 2:**
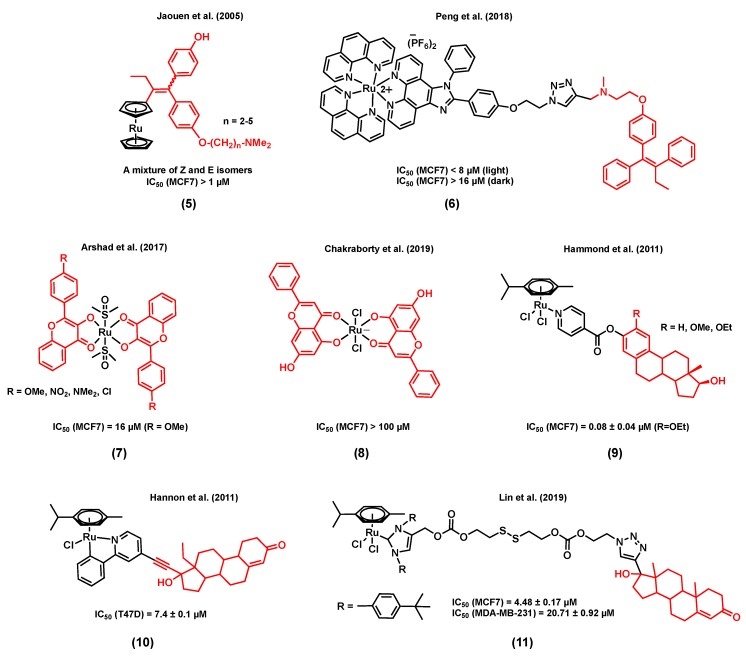
Ruthenium complexes bearing hormone receptor targeting moieties.

Accordingly, Jaouen et al. (2005) reported a series of ruthenocene-substituted tamoxifen derivatives, (5) (Figure 2), including alkyl chains of various lengths (*n* = 2–5) and investigated their in vitro cytotoxicity in both ER+ and TNBC cell lines [61]. Tamoxifen is known to compete with estrogens for the specific binding of estrogen receptors and, as a result, induce programmed cell death [62]. Notably, a slight activity was observed at 1 µM (% proteins/control ≈ 80) for the shortest alkyl chain complex (*n* = 2) in the ER+ cell line MCF7, an activity similar to that of the corresponding free ligand, whereas a slightly better cytotoxicity (% proteins/control ≈ 60–70) was noted for the derivatives with a longer alkyl chain (*n* = 3–5). No apparent cytotoxicity was observed when the TNBC cell line MDA-MB-231 was exposed to these ruthenium(II) complexes. Importantly, a much higher (>2 times) ERα relative binding affinity (RBA) was observed for the ruthenium complex bearing the shortest alkyl chain derivative (*n* = 2) when compared to that of its corresponding free ligand, demonstrating the receptor targeting potential of the ruthenium backbone [61]. Peng et al. (2018) reported an estrogen receptor-targeting ruthenium(II) polypyridyl photosensitizer, (6) (Figure 2), for the photodynamic therapy (PDT) of ER+ breast cancers [63], also bearing a tamoxifen derivative. The ruthenium polypyridyl backbone of the complex can serve as both a two-photon excited singlet oxygen-generating photosensitizer and a two-photon fluorescence probe for tracking the cellular uptake and localization of the drug candidate. On the other hand, the tamoxifen ligand linked to the ruthenium polypyridyl backbone through a triazole linker can provide efficient estrogen receptor targeting of ER+ breast cancer cells. Importantly, compound (6) displayed a significantly higher phototoxicity in ER+ breast cancer cells (MCF7) than in a triple negative cell line (MDA-MB-231), suggesting a non-negligible effect from tamoxifen on the internalization of the complex through its interaction with the multiple estrogen receptors found in MCF7 cells. The mode of action of this complex is believed to be associated with the generation of ^1^O_2_, causing damage to lysosomes, resulting in cell death. It is noteworthy that the phototoxicity of (6) was found to be significantly higher than that of a control compound (with no tamoxifen in its structure), but also higher than that of a mixture of the same control complex with tamoxifen (1:1 ratio), indicating a possible synergistic effect arising from the ruthenium and tamoxifen combination within a complex [63].

Other examples of estrogen receptor-targeting ruthenium species include complexes with substituted flavones as ligands, (7) (Figure 2), which were studied by Arshad et al. (2017) [64]. Flavones belong to a class of compounds called flavonoids, known to display different biological functions, including some antiestrogenic activity, due to their ability to bind estrogen receptors [65,66]. All the ruthenium-flavone complexes reported in this study displayed almost equal or slightly lower IC_50_ values in MCF7 breast cells compared to the corresponding flavones alone, suggesting a retained activity from the flavones upon coordination. It is also interesting to note that the lowest IC_50_ value in MCF7 cells (16 µM) was observed for a ruthenium complex that includes a flavone ligand bearing a methoxy substituent, known to inhibit DNA synthesis [64]. In another study, the potential modes of action of a ruthenium(III)-flavone (chrysin), complex (8) (Figure 2), was studied by Chakraborty et al. (2019). Results have demonstrated the ability of this compound to arrest the cell cycle and to induce apoptosis, following the upregulation of p53 and Bax and the downregulation of Bcl2, VEGF, and mTOR. The in vivo toxicity of (8) was also assessed by exposing rats to 250 to 1000 mg/kg doses of the complex. On Day 20, treatment-related mortality and body weight loss were observed when a 1000 mg/kg dose of (8) was used [67]. It is worth mentioning that none of the above publications on ruthenium-flavone complexes reported the potential interaction of the complexes with estrogen receptors.

It has been reported that the coordination of estrogens or androgens to an organometallic backbone can mediate hormone receptor targeting, facilitating the cellular uptake of the corresponding complexes [68,69]. For instance, a series of ruthenium(II) complexes with *N*-coordinated estradiol isonicotinates were reported by Hammond et al. (2011) (9) [70]. Their in vitro cytotoxicity in MCF7 cells was found to be considerable (IC_50_ values < 20 µM) with the highest activity being observed for the R = OEt derivative (IC_50_ = 0.08 ± 0.04 µM). Despite the promising cytotoxicity of these complexes, none of them showed any affinity for the estrogen receptor ERα. However, most of them were found to display a significant affinity, although to a lesser extent than their respective parent steroid, with the sex hormone binding globulin that transports steroid hormones in the blood and facilitates their cellular uptake by allowing their accumulation on the plasma membrane [70,71,72]. A steroid-conjugated (levonorgestrel) ruthenium(II) arene complex, (10) (Figure 2) [73], was reported by Hannon et al. (2011) to be 8-fold more active than cisplatin in T47D human breast cancer cells. The antiproliferative activities of free levonorgestrel and a control complex containing no steroid, Ru(*η^6^*-*p*-cymene)(ppy)Cl, were found to be much lower than the ruthenium bioconjugate complex. Theoretical DFT calculations on complex (10) showed that the metal center is distant enough from the lipophilic steroidal moiety to allow a possible interaction between the ruthenium and biomolecules such as N-nucleophiles, more specifically 9-ethylguanine (9-EtG), following the replacement of the chloride ligand with the nucleophile. Finally, ESI-MS analysis data also showed experimental evidence for the possible formation of a 9-EtG monoadduct, resulting from the incubation of 9-ethylguanine with (10). In another study, Lin et al. (2019) reported a ruthenium(II) N-heterocyclic carbene complex (Ru-NHC) conjugated to a 17α-ethynyl testosterone (Te structure very similar to progesterone) through a disulfide linkage to generate a new complex, Ru-NHC-S-S-Te, (11) (Figure 2) [74]. The cytotoxicity of (11) was studied in MCF7 (PR+) and MDA-MB-231 (PR-) cell lines, and was also compared with that of the original Ru-NHC complex as a control (with no steroid conjugated moiety). The IC_50_ value of (11) (4.48 ± 0.17 µM) was found to be about twice as low as that of Ru-NHC (10.54 ± 0.34 µM) in MCF7 cells. However, when MDA-MB-231 cells were treated with the complexes, an opposite trend was observed as a lower IC_50_ value was noted for Ru-NHC (14.18 ± 1.01 µM) when compared with that of (11) (20.71 ± 0.92 µM). The mode of action of (11) is associated with blocking the cell cycle progression and inducing cell apoptosis. Moreover, compound (11) showed a lower cytotoxicity in normal breast cells, Hs578Bst (IC_50_ = 37.36 ± 1.89 µM), compared with that of Ru-NHC in the same cell line (IC_50_ = 11.42 ± 1.12 µM). An ICP-MS analysis showed significantly higher ruthenium cellular levels in MCF7 cells treated with (10) compared to those treated with Ru-NHC. However, the ruthenium accumulation in the MDA-MB-231 cell line was found to be only slightly different between the two compounds. Taken together, cytotoxicity and cellular uptake studies suggest that the steroid moiety acts as a targeting unit to PR+ tumor cells. The in vivo antitumoral activity of (11) and Ru-NHC was also assessed in a nude mice MCF7 xenograft model. A slight decrease in the tumor volume of mice treated with (11) was noted, whereas the size of the tumor volume of the ones treated with Ru-NHC remained unchanged. Moreover, mice treated with (11) survived for a longer time than mice from the control group, whereas mice treated with Ru-NHC died prior to the ones from the control group, demonstrating the effect of the ligand on the toxicity of ruthenium complexes [74].

### 2.3. Other Ruthenium Complexes for the Treatment of Hormone Receptor Positive Breast Cancers

**Figure 3:**
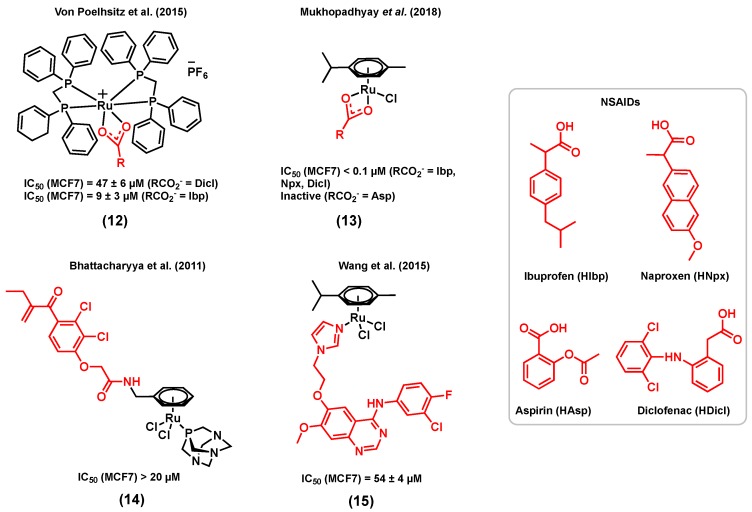
Ruthenium complexes bearing bioactive ligands.

Whereas examples of ruthenium complexes bearing ligands that have a specific target in hormone receptor positive breast cancers are limited, several complexes bearing other types of bioactive ligands with different targets were also found to display a considerable activity for hormone receptor positive cancers. For example, several reports have demonstrated that nonsteroidal anti-inflammatory drugs (NSAIDs) have promising anticancer properties, making them suitable ligands for the design of multifunctional anticancer ruthenium complexes [75,76,77]. The main mode of action of NSAIDs in different cancer types, including breast cancer, is believed to be associated with the inhibition of the cyclooxygenase (COX) enzyme [75,78]. The most obvious consequence of the overexpression of COX enzymes, more specifically COX-2, is the increased production of inflammatory prostaglandins (PG), mediators that may contribute to carcinogenesis, stimulate cancer cell proliferation, and mediate immune system suppression [79]. Furthermore, several studies suggest that COX-2 inhibitors such as NSAIDs might not only play a role in the treatment of breast cancer, but also in its prevention [80]. For instance, the in vitro cytotoxicity of two cationic ruthenium(II) dppm (diphenylphosphinomethane) complexes (12), respectively bearing diclofenac (Ru-Dicl) and ibuprofen (Ru-Ibp) (Figure 3) in MCF7 breast cancer cells, was reported by Von Poelhsitz et al. (2015) and compared with that of a ruthenium complex control (with no bioactive ligand), *cis*-[RuCl_2_(dppm)_2_], and cisplatin [81]. Both ruthenium complexes displayed a higher cytotoxicity (IC_50_ = 47 ± 6 µM for Ru-Dicl, MCF7; IC_50_ = 9 ± 3 µM for Ru-Ibp, MCF7) than that of the ruthenium complex control (IC_50_ = 191 ± 13 µM, MCF7), highlighting the importance of the NSAID ligand on the anticancer activity of the compounds. Moreover, the IC_50_ value of Ru-Ibp was found to be significantly lower than that of cisplatin (IC_50_ = 34 ± 4 µM, MCF7) [81]. It is worth mentioning that the potential COX inhibition activity of the compounds discussed above was not studied. More recently, Mukhopadhyay et al. (2018) reported a series of ruthenium(II) cymene complexes bearing different NSAID ligands, (13) (Figure 3), including diclofenac and ibuprofen, but also naproxen (Npx) and aspirin (Asp) [82]. Except for the Ru-Asp complex, which was found inactive against MCF7 cells, all complexes displayed a lower IC_50_ value (<0.1 µM) than their corresponding free NSAID (>80 µM) in this cell line. Furthermore, the COX inhibitory activity of the complexes was investigated, and all complexes showed higher in vitro COX inhibition than that of their corresponding free NSAID. Notably, the Ru-Ibp and Ru-Asp complexes could inhibit the activity of the enzyme more significantly than the Ru-Npx and Ru-Dicl species. The COX inhibition by Ru-Npx and Ru-Dicl and their corresponding NSAID was further investigated by a docking simulation. Both complexes exhibited significantly higher binding affinities than the corresponding free ligands naproxen and diclofenac towards COX-2, which could be due to the higher affinity and to the more extensive different non-bonding interactions of the ruthenium species with proximal amino acid residues of proteins, H-bonding interactions, and other non-bonding interactions such as halogen and pi–pi stacking interactions. Although this study suggests a potential for metal-NSAID complexes to target COX enzymes, the lack of stability of the reported complexes in DMSO and DMSO/water mixtures [82] prevents one from drawing conclusions about their potential multitargeting properties.

Another interesting example of a type of ruthenium(II) complex bearing an enzyme inhibitor was reported by Bhattacharyya et al. (2011), discussing a bifunctional ruthenium species of ethacrynic acid, a glutathione S-transferase (GST) inhibitor, (14) (Figure 3), and its in vitro cytotoxicity in MCF7 cancer cells [83]. Complex (14) is an analogue of the ruthenium arene complex RAPTA, which was previously reported for its promising anticancer potential in different cancer cell lines and its notable activity with regard to reducing the number and weight of solid metastases in vivo [32,84]. GSTs have multiple biological functions such as cell protection against oxidative stress and several toxic molecules. Because cancer cells overexpress GSTs, they can develop multifactorial drug resistance, making GST an efficient target for cancer therapy [85]. Among the members of the GST family, GSTP1-1, which catalyzes the conjugation of reduced glutathione (GSH) with a broad range of substrates including chemotherapeutic agents, has been linked to drug resistance and is frequently overexpressed in drug-resistant cell lines [86]. Exposure of MCF7 breast cancer cells to 20 µM of (14) led to a 10% reduction in cell viability after 24 h, whereas a more considerable reduction of 30% was noted after 72 h. Since reactive oxygen species (ROS) generation is one of the known modes of action of ethacrynic acid, ROS levels in cancer cells treated with (14) were also measured after 24 h and 72 h, resulting in significantly higher levels of ROS after 72 h. Due to its delayed cytotoxicity and ROS generation, compound (14) is believed to first interact with GSTP1-1, disrupting the apoptosis inhibition elicited by this enzyme, followed by the release of the metal fragment and the induction of cytotoxicity via a multiple mechanism pathway [83].

The epidermal growth factor receptor (EGFR) is also a coveted target for cancer therapeutics. Part of the receptor tyrosine kinase (RTK) family, EGFR is overexpressed in a broad range of human cancer cells, including breast cancer cells, making it a potential target for the development of new anticancer agents for breast cancer therapy [87]. The extracellular ligand-binding region of the EGFR or the intracellular tyrosine kinase region can be targeted by specific anticancer agents, which may interfere with the signaling pathways that modulate mitogenic and other cancer-promoting responses such as cell motility, cell adhesion, invasion, and angiogenesis [88]. 4-anilinoquinazoline derivatives are examples of antitumor agents for which the mode of action is to inhibit the tyrosine kinase activity of EFGR via competitive binding at the ATP site of the enzyme, resulting in cancer cell growth inhibition [89]. Wang et al. (2015) reported a series of ruthenium(II) cymene complexes of 4-anilinoquinazoline derivatives that showed dual-targeting properties, including a significant inhibitory activity of EGFR and a high affinity with DNA via a minor groove binding mode of interaction in different types of cancers including breast cancer [90]. In this series of complexes, the most notable results were obtained for (15) (Figure 3) for which the EGFR inhibitory activity (IC_50_ = 66.1 ± 11 nM) was very close to that of the corresponding 4-anilinoquinazoline ligand (IC_50_ = 60.2 nM) and higher than that of gefitinib, a well-known EGFR inhibitor (IC_50_ = 94.0 nM). The EGFR inhibitory activity of this complex was also supported by results obtained from a docking simulation. However, this study was performed for the aqua version of (15), as it was found to readily undergo hydrolysis in aqueous solutions. This result suggests that the introduction of ruthenium does not eradicate the activity of the 4-anilinoquinazoline ligand towards the EGFR. The presence of the chloride ligand in this class of complexes was found to be important for maintaining an EGFR inhibitory activity, which was found to be correlated to the hydrolysis potential of the compound, often considered as an essential step to activate metal-based complexes towards biomolecules [90]. The cytotoxicity of (15) was assessed in cancer cells, including human MCF7 breast cancer cells, either in the presence or in the absence of EGF (100 ng/mL), in order to evaluate the effect of blocking the signal transduction of the EGF on the inhibitory potency of the tested complexes. Complex (15) displayed a moderate cytotoxicity towards MCF7 cells (IC_50_ = 54 ± 4 µM) in the absence of EGF but was found to be inactive when exogenous EGF was added (IC_50_ > 100 µM). This result indicates that EGFR inhibition may not be the only mechanism of action for this complex, and other modes of action such as DNA interaction are likely to occur, which, however, may not be efficient enough to compensate for the effect of added EGF.

## 3. Ruthenium Complexes for the Treatment of Triple Negative Breast Cancer (TNBC) 

Since triple negative breast cancers do not respond to hormonal therapy, the commonly used anticancer drugs for hormone receptor positive breast cancers, such as P450 enzyme inhibitors or estrogen receptor targeting molecules, are not appropriate candidates to act as ligands in the structure of the ruthenium complexes for the treatment of this cancer. However, other types of anticancer agents could allow the preparation of ruthenium complexes with different cellular targets and thus improve their anticancer activity in TNBC or/and prevent the development of cancer cell resistance. In this section, several ruthenium complexes with remarkable anticancer activities in TNBC are discussed. Although the focus of this section is on multitargeting approaches, due to the importance of finding novel efficient drugs for the treatment of aggressive TNBC, other successful examples of ruthenium complex drug candidates are briefly presented, regardless of if their ligands are biologically active.

### 3.1. Ruthenium Complexes Bearing Nonsteroidal Anti-Inflammatory Drugs (NSAIDs)

**Figure 4:**
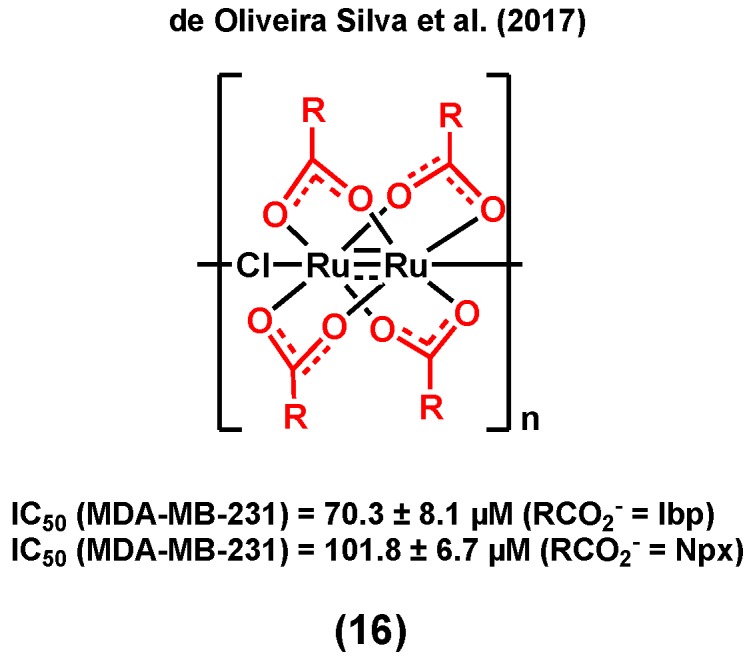
Ruthenium complexes bearing nonsteroidal anti-inflammatory drugs (NSAIDs).

It is noteworthy that NSAIDs may not only induce an anticancer activity in hormone receptor positive breast cancers, but also in TNBC, making them versatile moieties for the design of ruthenium species for breast cancer treatment. de Oliveira Silva et al. (2017) reported diruthenium(II,III) metallodrugs, (16) (Figure 4), of ibuprofen (Ru-Ibp) and naproxen (Ru-Npx) encapsulated into intravenously injectable solid–polymer–lipid nanoparticles (Ru-NSAID-SPLNs), which were prepared from a combination of two lipids (myristic acid and ethyl arachidate ester) [77]. The in vitro cytotoxicity of both dimeric metallodrugs was first studied in a triple negative breast cancer cell line, MDA-MB-231, and compared with that of ibuprofen and naproxen alone, and that of a ruthenium complex used as a control (which does not contain any bioactive ligand, [Ru_2_(O_2_CCH_3_)_4_Cl]). Although all observed IC_50_ values were found to be very high (>200 µM), the cytotoxicities of both dimeric metallodrugs, Ru-Ibp and Ru-Npx, were found to be higher than that of their corresponding parent drug, ibuprofen and naproxen, and that of the control ruthenium complex, suggesting that neither the diruthenium core nor the NSAID ligand alone is responsible for the observed anticancer activities. The encapsulation of the metallodrugs or the NSAIDs with SPLNs resulted in a significant enhancement of their anticancer activity. A higher cytotoxicity was observed for the Ru-NSAID-SPLNs compared to the NSAID-SPLNs, suggesting a contribution from the metal in both cases. Notably, a higher cytotoxicity was noted for Ru-Ibp-SPLNs in MDA-MB-231 cells (IC_50_ = 70.3 ± 8.1 µM) than for Ru-Npx-SPLNs (IC_50_ = 101.8 ± 6.7 µM) in the same cell line. It was postulated that the reported SPLN formulation can promote the cellular uptake of metallodrugs and, as a result, improve their anticancer potential [77].

### 3.2. Ruthenium Complexes Bearing a PARP Inhibitor

**Figure 5:**
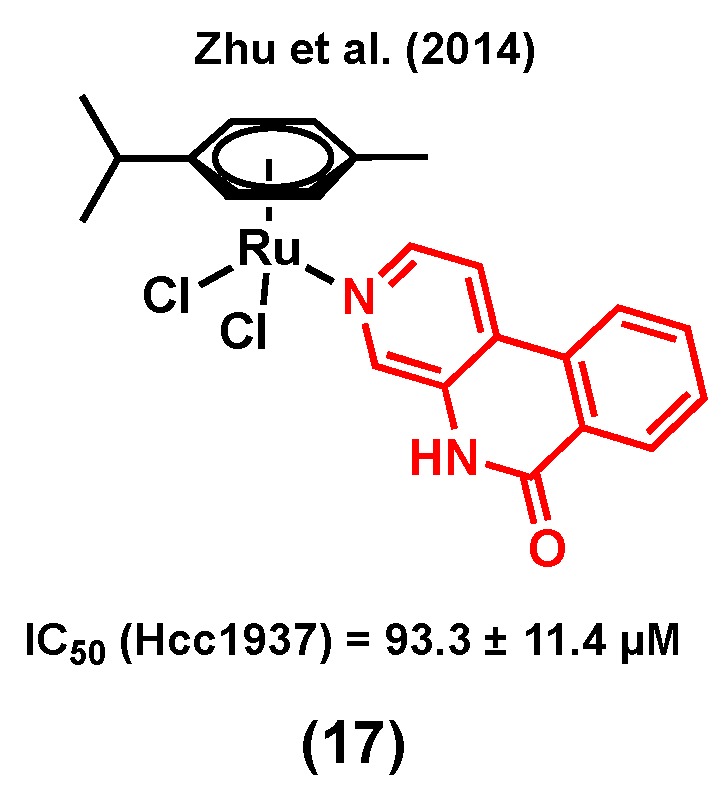
A ruthenium complex bearing Poly (ADP-ribose) polymerase (PARP) inhibitor.

Poly (ADP-ribose) polymerase (PARP) is a 17-member protein superfamily that has a well-established role in the DNA repair processes. The polymerization of ADP-ribose moieties to target proteins is catalyzed by PARPs using NAD^+^ as a substrate for which nicotinamide is a by-product of the process [91]. It is of interest that nicotinamide and its analogues were found to be a weak PARP inhibitor by acting as competitive inhibitors of the PARP substrate NAD^+^. Because PARP inhibitors interrupt the DNA repair processes and sensitize cells to DNA damaging agents, they are considered as promising anticancer candidates either as single agents or in combination with other anticancer drugs [91]. It is worth mentioning that PARP inhibitors are emerging as some of the most promising targeted therapeutics to treat TNBC [92], making them suitable candidates for the design of multitargeting ruthenium complexes for the treatment of this type of breast cancer. Notably, Zhu et al. (2014) developed ruthenium(II) arene anticancer complexes based on the PARP-1 inhibitor [93]. Interestingly, the coordination of this PARP inhibitor to ruthenium led to a more water-soluble species (solubility = 0.49 mM for (17) vs 0.12 mM for the free ligand) [93]. The resulting complex showed a higher in vitro cytotoxicity than its corresponding free PARP-1 inhibitor in different human cancer cell lines. Importantly, complex (17) (Figure 5) was found to be more cytotoxic in triple negative breast cancer Hcc1937 cells (IC_50_ = 93.3 ± 11.4 µM) than in noncancerous MRC-5 cells (IC_50_ = 143.0 ± 6.3 µM). It worth noting that RAPTA-C, a complex used as a control (with no PARP-1 inhibitor), did not induce any change in the viability of Hcc1937 cells, even at high concentrations (IC_50_ > 500 µM), highlighting the important contribution from the inhibitor in the observed activity of complex (17). The Ru-PARP inhibitor complex showed slightly better PARP inhibitory properties compared to the corresponding free inhibitor (IC_50_ of PARP-1 inhibition (µM): 0.32 for (17) vs 0.41 for the free ligand), and DNA-binding was also reported to be involved in its mode of action, suggesting that complex (17) would have multitargeting properties.

### 3.3. Ruthenium Complexes Bearing an Aerobic Glycolysis Inhibitor

**Figure 6:**
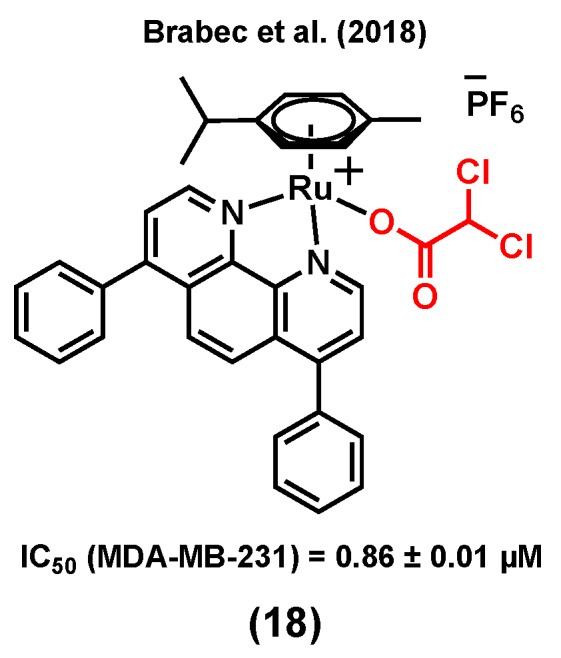
A ruthenium complex bearing dichloroacetato (DCA).

Unlike healthy tissues, tumors undergo aerobic glycolysis, a well-known metabolic reprogramming of cancer cells to sustain cell proliferation [94]. It has been reported that dichloroacetato (DCA) can inhibit this process, which is essential for cancer cells to produce energy in order to survive in the hypoxic environment of rapidly growing malignant tumors [95,96,97,98]. Brabec et al. (2018) reported a ruthenium(II) arene complex of DCA, (18) (Figure 6), with considerable cytotoxicity and antimetastatic properties in MDA-MB-231 cells [99]. The IC_50_ value of (18) in the MDA-MB-231 cell line was found to be 0.86 ± 0.01 µM (vs 56.0 ± 5.0 µM for cisplatin), whereas higher IC_50_ values were obtained when noncancerous cells HEK-293 (9.4 ± 0.5 µM) and primary skin fibroblasts (>50 µM) were treated with the complex. Compound (18) could reduce migration, invasion, and re-adhesion of TNBC cells, indicative of potential antimetastatic properties for this species. The antimetastatic properties of (18) were found to be associated with the ability of the compound to suppress matrix-metalloproteinase (MMP-9) activity and/or production, which is an important factor involved in the migration and adhesion processes [99,100]. Furthermore, compound (18) could slightly inhibit glycolysis in MDA-MB-231 cells, whereas cisplatin could not significantly impact glycolysis in this cell line. However, it is worth mentioning that osmium analogues of (18) showed more promising anticancer properties, such as a higher glycolysis inhibitory activity [99].

### 3.4. Ruthenium Complexes Bearing Gallic Acid

**Figure 7:**
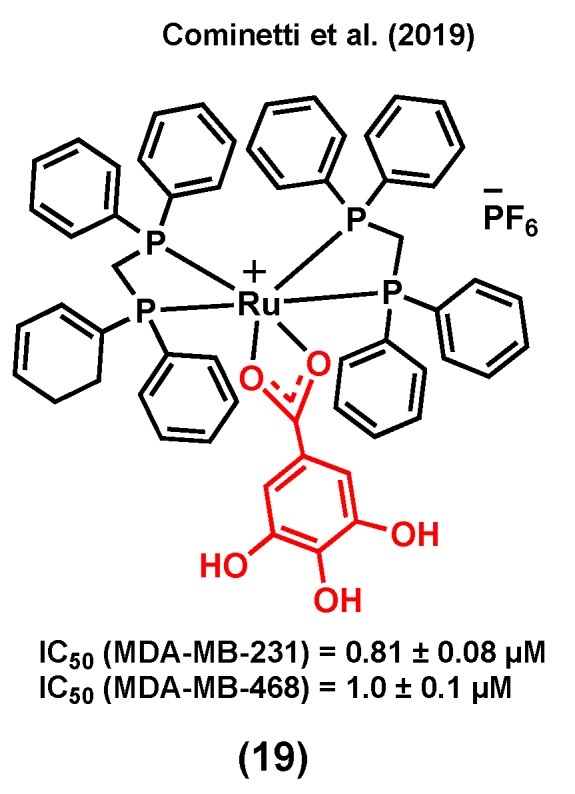
A ruthenium complex bearing gallic acid (GA).

Gallic acid can induce cell death by activating several signaling pathways in different cancer types such as breast, prostate, and lung [101]. Cell cycle arrest, and as a result, apoptosis, are possible mechanisms responsible for the anticancer activity of this compound [101,102]. A ruthenium(II) complex of gallic acid (GA), (19) (Figure 7), was synthesized by Cominetti et al. (2019) and its anticancer potential evaluated in triple negative breast MDA-MB-231 and MDA-MB-468 cancer cells, and in breast MCF-10A noncancerous cells [103]. Complex (19) exhibited a higher cytotoxicity in the two triple-negative cancer cell lines (IC_50_ = 0.81 ± 0.08 µM, MDA-MB-231; IC_50_ = 1.00 ± 0.10 µM, MDA-MB-468) than in MCF-10A noncancerous cells (IC_50_ = 5.82 ± 0.33 µM). The coordination of GA to ruthenium did not only lead to an improvement of the water solubility of its parent molecule, *cis*-[RuCl_2_(dppe)_2_] (which could not be tested due to its poor solubility in cell growth medium), but also led to a compound with a significantly increased cytotoxicity compared to that of GA (IC_50_ > 150 µM in both cancer cell lines). It is worth mentioning that the transferrin protein was found to play an important role in the internalization and cytotoxicity of (19). It was previously reported that some ruthenium species might have the ability to mimic iron to enter cancer cells via transferrin receptors, which are usually overexpressed in cancer cells compared to normal cells [103,104]. Accordingly, when concentrations of apo-transferrin were increased, while maintaining the (19) concentration constant, a significant drop in cancer cell viability was observed. However, under the same conditions, the viability of MCF-10A cells was not significantly altered, suggesting that ruthenium species are possibly more selective towards cancerous cells because of their higher levels of transferrin receptors [103].

### 3.5. Ruthenium Complexes Bearing Lapachol

**Figure 8:**
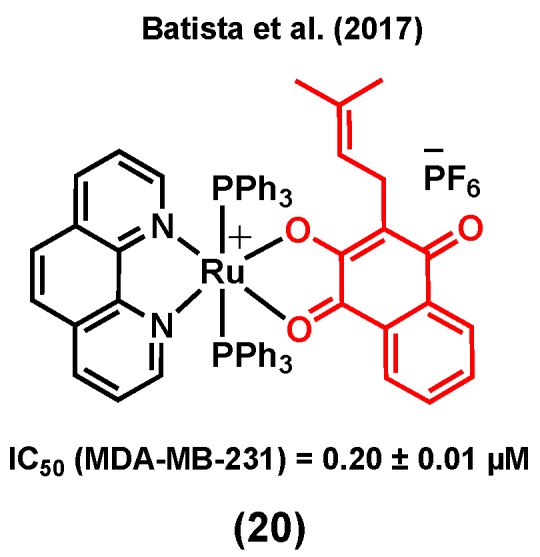
A ruthenium complex bearing lapachol.

Lapachol is a naturally occurring 1,4-naphthoquinone with known cytotoxicity and antimetastatic properties [105]. Its anticancer mode of action is known to mainly be due to its capacity to interact with topoisomerases and to generate reactive oxygen species (ROS) within cancer cells [106]. Batista et al. (2017) reported ruthenium(II) complexes of lapachol, (20) (Figure 8) and investigated their in vitro cytotoxicity in TNBC MDA-MB-231 cells [107]. Results showed a notable improvement in the anticancer activity of lapachol upon complexation to ruthenium (IC_50_ = 0.20 ± 0.01 μM for (20) vs IC_50_ > 100 μM for lapachol). Besides, it was suggested that the main mode of action of this complex would most likely not involve DNA binding, as only very weak DNA interactions were observed for (20) [107].

### 3.6. Ruthenium Complexes Bearing Biotin

**Figure 9:**
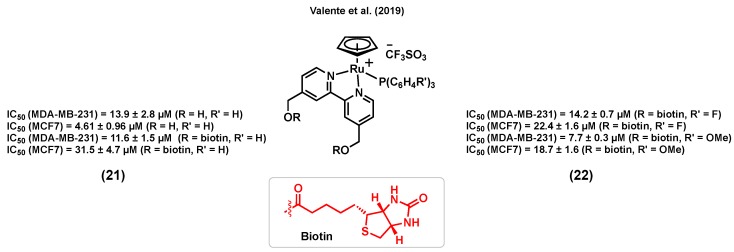
Ruthenium complexes bearing biotin.

Vitamin–drug conjugates have attracted increased attention for cancer therapy in the last years as they can lead to an enhancement in the cancer cell uptake of some drugs due to the overexpression of vitamin receptors at their surface [108,109]. For instance, biotin (vitamin B7) is a promising candidate for exploiting this strategy due to its potential cancer cell selectivity resulting from its receptor-mediated uptake [110]. Notably, a sodium-dependent multivitamin transporter (SMVT) is overexpressed in different cancer cell lines, including breast cancer, making it an appropriate target for the selective treatment of this disease [111]. Accordingly, Valente et al. (2019) reported a ruthenium(II) cyclopentadiene complex bearing biotin, (21) (R = biotin), with a considerable in vitro cytotoxicity in MDA-MB-231 breast cancer cells (IC_50_ = 11.6 ± 1.5 μM) [112] (Figure 9). The study showed that (21) (R = biotin) could block the activity of the ABC transporter, P-glycoprotein (P-gp), which is known to play an important role at inducing multidrug resistance (MDR) in several cancer cells [113]. It is worth noting that the inhibition of P-gp was not observed for a biotin-free analogue of the complex (R = H), demonstrating the importance of this conjugation to biotin [112]. An overall toxicity assessment of (21) (R = biotin) on the embryonic development of zebrafish revealed a tolerance up to 1.17 mg/L for this complex, with morphologic lesions such as curved spine/tail malformation, yolk sac and pericardial sac edema, cranial malformation, and underdeveloped eyes being observed when embryos were exposed to a concentration of 2.18 mg/L or higher [112]. In another publication, Valente et al. (2019) also reported analogous biotinylated ruthenium(II) cyclopentadiene complexes for breast cancer therapy, bearing a substituted triphenylphosphine [111]. Among these species, complexes (22) (Figure 9) were found to be generally more cytotoxic to MDA-MB-231 cells than MCF7 cells, with a more considerable activity being observed for the species bearing an electron donating substituent on the phosphine aryl groups (IC_50_ = 7.7 ± 0.3 μM, R′ = OCH_3_) than for the analogous version of the complex bearing an electron withdrawing substituent (IC_50_ = 14.2 ± 0.7 μM, R′ = F). To evaluate the ability of the complexes to interact with SMVT, the interaction of the species with avidin, a tetrameric glycoprotein with high specificity and affinity to biotin, was studied [114]. Importantly, the biotinylated complexes showed a significant affinity to avidin, although to a lesser extent than that of biotin alone, whereas nonbiotinylated analogues of these compounds did not interact with this protein, indicating that the complexes bearing the vitamin can potentially target SMVT. The in vivo toxicity evaluation of some of the complexes on the development of zebrafish embryos revealed that the biotinylated species (22) caused less severe toxic effects (major lesions were yolk sac and pericardial sac edemas) compared to the nonbiotinylated complexes (necrosis/cell lysis), suggesting that the targeting approach could lead to an increased in vivo tolerability. Moreover, these complexes were reported to be mainly retained within the membrane of cancer cells (>90% for MDA-MB-231) and to be able to inhibit the formation of colonies (loss of adhesive interactions), an indication of the antimetastatic behavior potential of these species [111].

### 3.7. Other Ruthenium Complexes for the Treatment of TNBC

**Figure 10:**
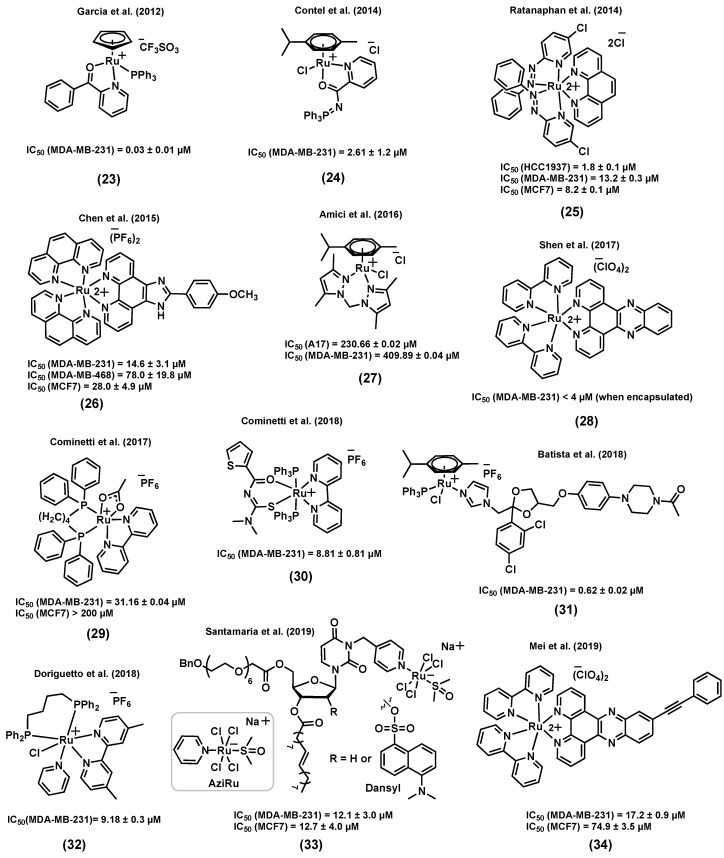
Other ruthenium complexes for triple negative breast cancer (TNBC) treatment.

Several ruthenium complexes bearing ligands that were not previously reported to display a biological activity were found to be highly active in vitro and/or in vivo against aggressive TNBC. For instance, Garcia et al. (2012) reported a ruthenium(II) cyclopentadiene species (23) (Figure 10), TM90, for which the in vitro cytotoxicity was found to be particularly high (IC_50_ = 0.03 ± 0.01 μM) in MDA-MB-231 cells, and considerably higher than that of cisplatin (IC_50_ = 39.0 ± 5.0 μM) in the same cell line [115]. The important stability of this complex in a cell growth medium containing 2% DMSO (used as a vehicle) was reported a few years later by Garcia et al. (2017) [116]. Interestingly, it was found that (23) could form an adduct with human serum albumin (HSA). The coincubation of the complex with different concentrations of HSA showed no significant change in the observed cytotoxicity, suggesting that the interaction of (23) with HSA does not inactivate the complex and could likely facilitate its distribution and delivery to cancer cells [116]. Moreover, MDA-MB-231 cells exposed to (23) showed a major population of necrotic cells and a smaller population of apoptotic cells, indicating that necrosis could be the main cell death mechanism caused by this species. An investigation of the in vivo antitumor activity of (23) on an MDA-MB-231 tumor in female athymic nude mice demonstrated the superior ability of this complex at suppressing tumor growth over time. Furthermore, mice treated with (23) showed a significantly increased lifetime after surgical removal of the tumor compared to those that were not treated. Unlike cisplatin, which induced significant body weight loss, no apparent change was observed between mice treated with (23) and the control group over time, showing that this compound did not affect the well-being of the animals.

In another study, Contel et al. (2014) reported a series of ruthenium(II) arene complexes of nontoxic iminophosphorane (IM) ligands with a promising in vitro and in vivo anticancer potential in TNBC [117]. Metal complexes of IM displayed a high in vitro cytotoxicity in a variety of human cancer cell lines with different degrees of selectivity and pathways other than DNA interaction, such as mitochondrial production of ROS and inhibition of PARP-1 proteins [118,119,120]. Compound (24) (Figure 10) was found to be highly water-soluble and to display a considerable cytotoxicity against several cisplatin resistant cell lines. Notably, the cytotoxicity of (24) in MDA-MB-231 was found to be much higher (IC_50_ = 2.61 ± 1.2 μM) than that of cisplatin (IC_50_ = 131.2 ± 18 μM) in this cell line. However, this complex was not found to be selective towards cancer cells, as it was found to also be highly cytotoxic in a noncancerous cell line, HEK-293T (IC_50_ = 2.8 ± 0.2 μM). The mode of action of this complex was reported to take place through canonical- or caspase-dependent apoptosis, whereas DNA interaction and protease cathepsin B inhibition were not found to be likely to take place. Moreover, NOD.CB17-Prkdc scid/J mice were used as an in vivo model in which MDA-MB-231 tumor cells were injected. A significant tumor reduction of 56% was reported after a 28 day-treatment (14 doses of 5 mg/kg of (24) every other day) with low systemic toxicity and preferential accumulation in the breast tumor tissues compared to other organs such as kidney and liver, suggesting the high in vivo efficacy of this complex [117].

It has been reported that ruthenium-based agents can be promising anticancer candidates to treat BRCA1-mutant breast cancers, frequently associated with TNBC [121,122]. The BRCA1 gene responds to DNA damage by being involved in cellular pathways for DNA repair, mRNA transcription, cell cycle regulation, and protein ubiquitination [123,124]. The role of BRCA1 is also to regulate chemotherapy-induced DNA damage [124]. Ratanaphan et al. (2014) reported ruthenium(II) complexes that showed a promising cytotoxicity in a BRCA1 defective TNBC cell line, HCC1937 [121]. Importantly, complex (25) (Figure 10) induced significantly more cytotoxicity in BRCA1 defective-HCC1937 cells (IC_50_ = 1.8 ± 0.1 µM) than in the BRCA1 wild-type cell lines MDA-MB-231 (IC_50_ = 13.2 ± 0.3 µM) and MCF7 (IC_50_ = 8.2 ± 0.1 µM), suggesting that the higher sensitivity of the BRCA1 defective breast cancer cells to (25) might be due to the inability of the dysfunctional BRCA1 to repair ruthenium-induced DNA damage. Besides, cell exposure to (25) demonstrated a higher degree of cytotoxicity than cisplatin against all three cell lines. Upon internalization in HCC1937 cells, (25) was found to be mainly located in the nuclear fraction after 12–48 h. Moreover, a significant inhibition in the G2/M phase of the cell cycle, an increased induction of apoptotic cells, an upregulation of p53 mRNA and a downregulation of BRCA1 mRNA were observed in breast cancer cells treated with (25) [121].

In another example of ruthenium complexes for the treatment of TNBC, Chen et al. (2015) identified a ruthenium(II) complex, (26) (Figure 10), that could act as a potent antimetastatic agent and metal-based chemosensitizer towards MDA-MB-231 cells [125]. Complex (26) induced a higher cytotoxicity in TNBC cell lines (IC_50_ = 14.6 ± 3.1 µM, MDA-MB-231; 78.0 ± 19.8 µM, MDA-MB-468) than in human normal kidney cells (IC_50_ = 143.9 ± 10.2 µM, HK-2), and its cytotoxicity is believed to be associated with transferrin-mediated endocytosis. A low-dose (1–2 µM) and short-term treatment of (26) inhibited the migration and invasion of MDA-MB-231 cells in a cytotoxicity-independent manner. Regulating the expression levels of metastatic regulatory proteins and inhibiting the secretion of vascular endothelial growth factor (VEGF) were suggested to be associated with the anticancer activity of (26) in MDA-MB-231 cells. A co-treatment of MDA-MB-231 cells with (26) and tumor necrosis factor–related apoptosis-inducing ligand (TRAIL) suggested that the ruthenium complex could potentiate TRAIL-induced apoptosis through intrinsic and extrinsic apoptotic pathways, indicating that this combined treatment could be a novel strategy to inhibit the growth and the metastatic potential of tumor cells and synergistically enhance TRAIL-induced apoptotic cell death [125].

Amici et al. (2016) reported a water soluble ruthenium(II) complex, (27) (Figure 10), with potent in vivo antitumor activity against TNBC [126], even though it displayed a very poor in vitro activity in TNBC cell lines (IC_50_ = 230.66 ± 0.02 µM, A17; 409.89 ± 0.04 µM, MDA-MB-231). As a reference, it is interesting to note that the complex was found to be much less cytotoxic than cisplatin (IC_50_ = 6.93 ± 0.14 µM, A17; 38.70 ± 0.03 µM, MDA-MB-231) but nevertheless more cytotoxic than NAMI-A (IC_50_ = 485.58 ± 0.02 µM, A17; 840.21 ± 0.03 µM, MDA-MB-231) in the same cell lines. Interestingly, only (27) resulted in the induction of cancer cell death by the activation of the apoptotic caspase-3 when compared to NAMI-A or cisplatin treatments. A female FVB/NCrl mice model with A17 cells was then used to study the in vivo antitumoral activity of (27). An intraperitoneal injection of (27) (52.5 mg/kg/day) or cisplatin (3 mg/kg/day), repeated 4 times at 3-day intervals, resulted in a decrease in tumor volume compared to that of untreated mice, whereas the same treatment with NAMI-A (52.5 mg/kg/day) was found to be less effective. NAMI-A and cisplatin treatments were associated with weight loss, whereas the body weight of the mice treated with (27) was not found to be significantly different than that of untreated mice, suggesting a low toxicity for this complex at the selected dose. The antitumor activity of (27) is believed to be associated with reduced regulatory T cells (T_reg_) infiltration and increased dendritic cells/macrophage recruitment into the tumor microenvironment [126].

Shen et al. (2017) reported a liposome-based nanodelivery system as a strategy to improve the anticancer potential of a ruthenium(II) complex of dipyridophenazine (dppz), (28) (Figure 10), against TNBC [127]. This liposome encapsulation strategy did not only improve the biodistribution and pharmacokinetics of the ruthenium complex, but could also provide a hydrophobic environment, helping the ruthenium species emit fluorescence light and, as a result, leading to nanoparticle tracking inside the body. The cell viability of MDA-MB-231 cells was studied upon exposure to liposomes alone, compound (28), and encapsulated-(28). The two former molecules did not notably change the viability of cancer cells; however, the encapsulated complex could significantly inhibit the cell viability (IC_50_ < 4 µM). Cell uptake studies demonstrated considerably higher ruthenium cellular uptake for the encapsulated complex (about 15-fold higher) than the complex itself. An athymic nude mice inoculated with MDA-MB-231 tumor cells was used to further study the in vivo anticancer potential of the species. A considerable tumor size shrinkage was observed for the mice treated with the encapsulated complex compared to those treated with the ruthenium complex, indicating that the liposome encapsulation is not only enhancing the in vitro cytotoxicity of the ruthenium complex but also its in vivo activity. It is worth mentioning that no apparent morphological changes were observed in tumor free mice treated with the encapsulated complex, suggesting potential selectivity for this system. DNA damage, cell cycle arrest, and apoptosis were reported to be possible modes of action of the liposome-encapsulated ruthenium complex (28) [127].

Cominetti et al. (2017) reported a series of biphosphine bipyridine ruthenium(II) complexes with considerable cytotoxicity and antimetastatic potential in TNBC [128]. From this series, compound (29) (Figure 10) showed the most significant anticancer activity. This complex could reduce the viability of MDA-MB-231 cells (IC_50_ = 31.16 ± 0.04 µM) to a greater extent than that of the ER+ breast cell line MCF7 (IC_50_ > 200 µM) and the noncancerous breast cell line MCF-10A (IC_50_ = 48.89 ± 0.09 µM), suggesting a possible selectivity towards triple negative cancers. Although this complex did not induce a high cytotoxicity at low concentration (20 µM), it could notably inhibit migration, adhesion, and invasion of MDA-MB-231 cells, likely due to the inhibition of the MMP-9 enzyme and the alteration of the cytoskeleton proteins responsible for the provision of the basic infrastructure for the maintenance of cell adhesion and motility [128].

Cominetti et al. (2018) also reported a ruthenium(II) complex of acylthiourea, (30) (Figure 10), which was also found to be active against TNBC tumor cells [129]. The inhibition of proliferation, migration, invasion, and adhesion was observed for cancer cells exposed to (30). The in vitro cytotoxicity of this compound in MDA-MB-231 cancer cells (IC_50_ = 8.81 ± 0.81 µM) was found to be higher than that in the noncancerous breast cell line MCF-10A (IC_50_ = 14.82 ± 2.50 µM). Furthermore, a change in morphology, induced apoptosis, DNA damage, and nuclear fragmentation were reported as possible modes of action for this complex. The in vivo toxicity of (30) was assessed using a mice model. At the doses administered intraperitoneally (50 and 300 mg/kg), this compound did not lead to a change in the weight of the animal compared to the control groups, which is indicative of a low toxicity [129].

Since azole compounds usually show a broad range of biological activities such as antifungal and anticancer properties because of their affinity to bind to biomolecules [130,131], Batista et al. (2018) reported a series of ruthenium(II) arene complexes tethering azole-containing ligands and studied their in vitro cytotoxicity and antimigration activity [132]. Although the azole-containing drugs used in this study are known as antifungal species, promising cytotoxicities against the TNBC cell line MDA-MB-231 were observed, more particularly for compounds for which ketoconazole was coordinated to the metal center, (31) (IC_50_ = 0.62 ± 0.02 μM) (Figure 10). Furthermore, human serum albumin (HSA) binding was reported as one of the possible modes of action for these compounds [132].

Doriguetto et al. (2018) reported a new series of ruthenium(II) diimine/phosphine complexes and tested their in vitro cytotoxicity in various cell lines [133]. For instance, complex (32) (Figure 10) showed a high cytotoxicity in the MDA-MB-231 cell line (IC_50_ = 9.18 ± 0.30 µM) compared to that in fibroblasts derived from normal skin (IC_50_ = 24.19 ± 3.02 µM), indicating a potential selectivity for this species. Complex (32) induced morphological changes and inhibited the size and number of colonies, suggesting an anti-clonogenic activity against MDA-MB-231 cells. Moreover, apoptosis was induced by (32) in MDA-MB-231 tumor cells in a concentration-dependent manner [133].

Some nucleolipidic ruthenium(III) complexes incorporated into a nanosystem have also been developed as a potential strategy for cancer therapy and have shown some promising anticancer potential in both ER+ breast and TNBC cancer cell lines [134,135]. For instance, Santamaria et al. (2019) reported nanosystems designed to improve the efficacy of nucleolipidic anticancer ruthenium(III) complexes for biomedical applications, and to deliver AziRu (Figure 10), a ruthenium(III) complex structurally inspired by the well-known drug candidate NAMI-A [135]. The ruthenium(III) complex, (33) (R = H) (Figure 10), was incorporated into a 1,2-dioleoyl-3-trimethylammoniumpropane chloride (DOTAP) nanocarrier and its cytotoxicity in breast cancer cells (ER+ and TNBC) was compared with that of AziRu (Figure 10) and cisplatin. Notably, in the TNBC cell line MDA-MB-231, the (33)/DOTAP (R = H) nanosystem proved to be more effective (IC_50_ = 12.1 ± 3 µM, corresponding to the effective metal concentration carried by the nanoaggregate, 15% mol/mol,) than cisplatin (IC_50_ = 19 ± 4 µM), in contrast to AziRu, which was found to be inactive. Importantly, (33)/DOTAP (R = H) did not induce a high cytotoxicity in non-cancerous MCF-10A cells (IC_50_ > 100 µM). A fluorescently-tagged analogue of complex (33) (R = dansyl) (Figure 10) was developed to further study the cellular uptake and accumulation of the compound. Although it was previously reported that a large extent of AziRu (about 80%) remained in the culture medium after incubation, large intracellular amounts of ruthenium (about 85% of the administered quantity) were found (mainly at the nuclear level) after treatment with (33)/DOTAP (R = dansyl). Furthermore, Bcl-2 down-regulation and autophagy were suggested to be involved in the mechanism(s) of action of this complex. An in vivo assessment of the antitumoral potential of (33) (R = H) was also performed using athymic nude mice bearing human BCC xenografts (although only using MCF7 cells inoculated into the nude mice). After the administration of (33)/DOTAP (R = H) at a 15 mg/kg (i.p.) dose, once a week for 28 days, the weight and volume of tumors were found to be significantly reduced. Also importantly, the treatment was well-tolerated in mice since no sign of toxicity was observed [135].

Finally, Mei et al. (2019) reported a class of ruthenium(II) phenazine derivatives (DPPZ) with interesting anticancer properties against TNBC cells [136]. The most promising results were obtained for compound (34) (Figure 10), which displayed a notable inhibitory activity against the proliferation (IC_50_ = 17.2 ± 0.9 µM), migration, and invasion of MDA-MB-231 cells. A structure-activity relationship analysis showed that the increased number of aromatic planar rings in the ligands can effectively enhance the antitumor activity of this series of complexes. Compound (34) was found to enter breast cancer cells and localize into the nucleus, which is indicative that the complex might induce DNA damage to cause cell apoptosis. Furthermore, an in vivo anticancer evaluation of (34) in the xenograft model of human MDA-MB-231 in zebrafish showed that the number of cancer cells was notably reduced compared with the control group, suggesting that (34) can effectively suppress the proliferation of TNBC cells in zebrafish. Moreover, a scarce number of MDA-MB-231 cells were found in the blood vessels of zebrafish, suggesting that (34) might inhibit the metastasis of the cancer cells in vivo [136].

## 4. Conclusions

In this review, we briefly report rationally designed ruthenium complexes bearing bioactive ligand(s) as potential candidates for the treatment of hormone receptor positive breast cancers and TNBCs. The bioactive ligands included in these complexes are known to have a broad range of molecular targets such as enzymes, hormone receptors, growth factors, etc. Some of these complexes could undergo multiple anticancer mechanisms or/and illustrate a synergistic effect. Cytotoxicities and antimetastatic activities can both be induced in breast cancer cells using these types of ruthenium complexes. As expected, the most promising cytotoxicities reported were noted for ruthenium (II) complexes, which is in line with the inert nature of ruthenium (III) species. Notably, ruthenium (II) arene and ruthenium (II) cyclopentadienyl complexes were found to display the most interesting activities, with IC_50_ values lower than 1 µM in some cases. The information presented in this review also revealed low IC_50_ values for several ruthenium species in TNBCs, which normally do not respond to currently used drugs, making these compounds attractive candidates for further investigation. Despite the fact that several publications have presented the in vitro activity of ruthenium complexes in breast cancer cells, fewer reports have presented results regarding the solubility, stability, and in vivo anticancer activity of these species. Moreover, the studies reported so far were not always performed under the same conditions (stock solution preparation, type of in vitro assay/in vivo model, duration of treatment, etc.), preventing a direct comparison of their overall bioactivity. We believe that this review can contribute to open new doors for further investigation in the development of novel Ru-based complexes for the treatment of the most frequently diagnosed cancer among women.

## Supplementary Materials

The following are available online, Table S1: Summary of the activity of the ruthenium complexes reviewed in this study.
