# Supplementary material for: Rationally Designed Ruthenium Complexes for Breast Cancer Therapy
Source: Molecules. 2020 Jan 9;25(2):265. doi: 10.3390/molecules25020265 (PMC7024301; doi:10.3390/molecules25020265)
Supplement: Supplementary file 1 [file molecules-25-00265-s001.pdf]

SUPPLEMENTARY INFORMATION

Rationally designed ruthenium complexes for breast cancer therapy

Golara Golbaghi, Annie Castonguay*

Organometallic Chemistry Laboratory for the Design of Catalysts and Therapeutics, INRS-Centre Armand-Frappier Santé Biotechnologie, Université du Québec, Laval, Canada

TABLE OF CONTENT

Table S1. Summary of the biological activity of the ruthenium complexes reviewed in this study S2-S6

References S7-S9

Table S1. Summary of the biological activity of the ruthenium complexes reviewed in this study.

		Bioactive ligand	In vitro IC_{50} (μM) values in breast cancer cells	In vivo antitumoral activity and/or in vivo toxicity
**1**	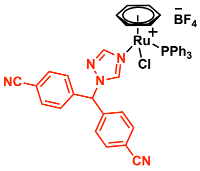	Aromatase inhibitor (letrozole)	>25 (MCF7)	N.A. [1]
**2**	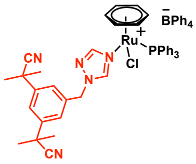	Aromatase inhibitor (anastrozole)	≈ 4 (MCF7 and T47D)	No toxicity (12.5 μM) on the development of zebrafish embryos. [2]
**3**	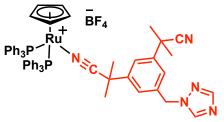	Aromatase inhibitor (anastrozole)	0.50 ± 0.09 (MCF7)<br>0.32 ± 0.03 (T47D)<br>0.39 ± 0.09 (MDA-MB-231)	No toxicity (at concentrations around its IC_{50} values) on the development of zebrafish embryos. [3]
**4**	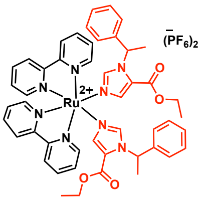	P450 enzyme inhibitor (etomidate)	N.A.	N.A. [4]
**5**	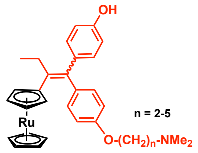	Steroid hormone receptor targeting molecules (tamoxifen derivatives)	>1 (MCF7)	N.A. [5]
**6**	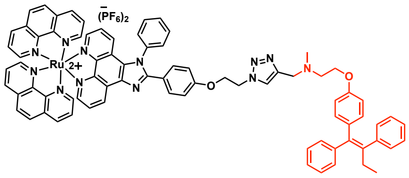	Steroid hormone receptor targeting molecule (tamoxifen)	<8 (light) (MCF7)<br>>16 (dark) (MCF7)	N.A. [6]
**7**	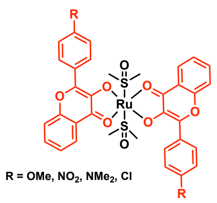	Steroid hormone receptor targeting molecule (flavone)	16.0 (MCF7) when R = OMe	N.A. [7]

**8**	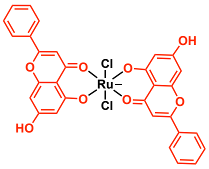	Steroid hormone receptor targeting molecule (flavone)	> 100 (MCF7)	Mortality and body weight loss in rats (1000 mg/kg, at Day 20). [8]
**9**	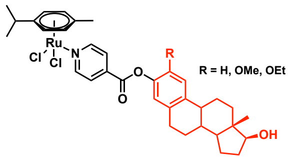	Steroid hormone receptor targeting molecules (estradiol isonicotinates)	0.08 ± 0.04 (MCF7) when R = OEt	N.A. [9]
**10**	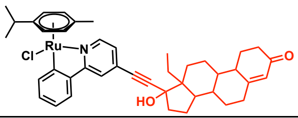	Steroid hormone receptor targeting molecule (levonorgestrel)	7.4 ± 0.1 (T47D)	N.A. [10]
**11**	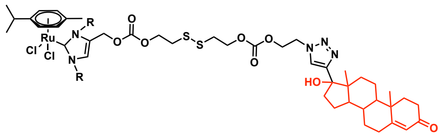	Steroid hormone receptor targeting molecule (17α-ethynyl testosterone)	4.48 ± 0.17 (MCF7)<br>20.71 ± 0.92 (MDA-MB-231)	Slight decrease in tumor volume (2 μmol/kg, every 4 days, 5 doses) in mice bearing MCF7 cells. [11]
**12**	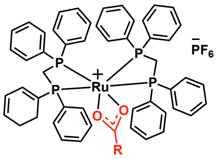	Nonsteroidal anti-inflammatory drugs (NSAIDs) (diclofenac, ibuprofen)	Ru-Dicl: 47 ± 6 (MCF7)<br>Ru-Ibp: 9 ± 3 (MCF7)	N.A. [12]
**13**	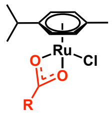	Nonsteroidal anti-inflammatory drugs (NSAIDs) (diclofenac, ibuprofen, naproxen, aspirin)	<0.1 (MCF7) except when RCOO$^-$ = aspirin which was inactive	N.A. [13]
**14**	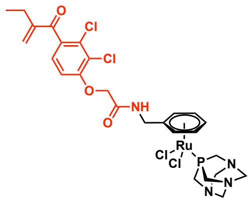	Glutathione S-transferase (GST) inhibitor (ethacrynic acid)	>20 (MCF7)	N.A. [14]
**15**	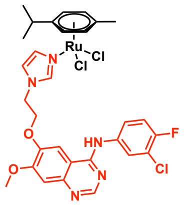	Epidermal growth factor (EGFR) inhibitors (4-anilinoquinazoline derivatives)	54 ± 4 (MCF7)	N.A. [15]

**16**	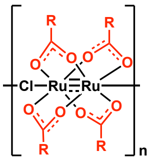	Nonsteroidal anti-inflammatory drugs (NSAIDs) (ibuprofen, naproxen)	Ru-Ibp-SPLNs: 70.3 ± 8.1 (MDA-MB-231)<br>Ru-Npx-SPLNs: 101.8 ± 6.7 (MDA-MB-231)	N.A. [16]
**17**	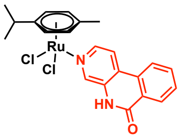	Poly (ADP-ribose) polymerase (PARP) inhibitor	93.3 ± 11.4 (Hcc1937)	N.A. [17]
**18**	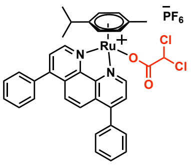	Aerobic glycolysis inhibitor (dichloroacetato)	0.86 ± 0.01 (MDA-MB-231)	N.A. [18]
**19**	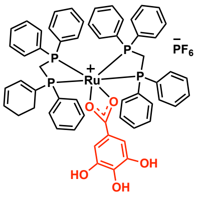	Cell cycle arrest inducer (gallic acid)	0.81 ± 0.08 (MDA-MB-231)<br>1.0 ± 0.1 (MDA-MB-468)	N.A. [19]
**20**	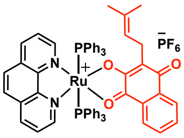	Topoisomerase-interacting and ROS-generating molecule (lapachol)	0.20 ± 0.01 (MDA-MB-231)	N.A. [20]
**21**	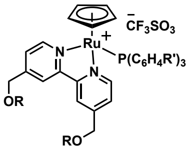	Biotin	11.6 ± 1.5 (MDA-MB-231)<br>31.5 ± 4.7 (MCF7)<br>when R = biotin and R′= H	Zebrafish tolerance up to 1.17 mg/L. Morphologic lesions such as curved spine/tail malformation, yolk sac and pericardial sac edema, cranial malformation and underdeveloped eyes were observed at 2.18 mg/L. [21]
**22**	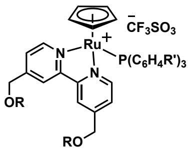	Biotin	14.2 ± 0.7 when R′= F and R = biotin<br>7.7 ± 0.3 when R′= OCH_3 and R= biotin (MDA-MB-231)<br>22.4 ± 1.6 when R′= F and R= biotin<br>18.7 ± 1.6 R′= OCH_3 and R= biotin (MCF7)	LC_{50} values (lethality for 50% of the embryos/larvae) on zebrafish (120 hpf, 1.83-2.35 mg/L). Moderate to severe yolk sac edema and pericardial sac edema were observed. [22]

**23**	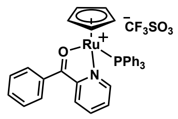	N.A.	0.03 ± 0.01 (MDA-MB-231)	Suppression of tumor growth (2.5 mg/kg/day, 10 days) in female athymic nude mice bearing MDA-MB-231 cells. No effect on the well-being of the animals. [23,24]
**24**	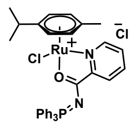	N.A.	2.61 ± 1.2 (MDA-MB-231)	Significant decrease (56%) in tumor volume (5 mg/kg, every other day, 14 doses) in NOD.CB17-Prkdc SCID/J mice bearing MDA-MB-23 cells. Low systemic toxicity. [25]
**25**	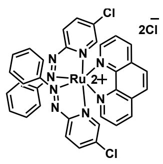	N.A.	1.8 ± 0.1 (HCC1937)<br>13.2 ± 0.3 (MDA-MB-231)<br>8.2 ± 0.1 (MCF7)	N.A. [26]
**26**	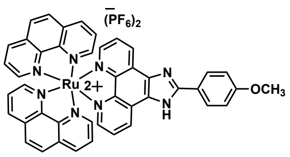	N.A.	14.6 ± 3.1 (MDA-MB-231)<br>78.0 ± 19.8 (MDA-MB-468)<br>28.0 ± 4.9 (MCF7)	N.A. [27]
**27**	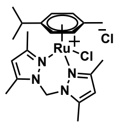	N.A.	230.66 ± 0.02 (A17)<br>409.89 ± 0.04 (MDA-MB-231)	Suppression of tumor growth (52.5 mg/kg, every 3 days, 4 doses) in female FVB/NCrl mice bearing A17 cells.<br>No apparent toxicity. [28]
**28**	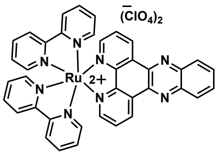	N.A.	<4 (when complex is encapsulated)	Suppression of tumor growth (5 mg/kg/week, 4 doses) (encapsulated complex) in athymic nude mice bearing MDA-MB-231 cells. No apparent toxicity. [29]
**29**	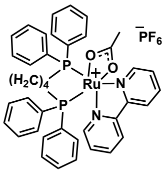	N.A.	31.16 ± 0.04 (MDA-MB-231)<br>>200 (MCF7)	N.A. [30]
**30**	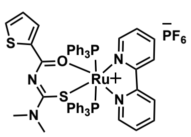	N.A.	8.81 ± 0.81 (MDA-MB-231)	Low toxicity (up to 300 mg/kg, 14 days) in a Swiss mice model. [31]

**31**	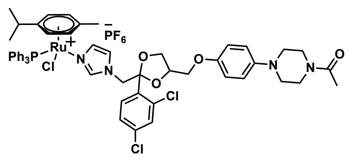	N.A.	0.62 ± 0.02 (MDA-MB-231)	N.A. [32]
**32**	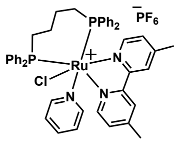	N.A.	9.18 ± 0.30 (MDA-MB-231)	N.A. [33]
**33**	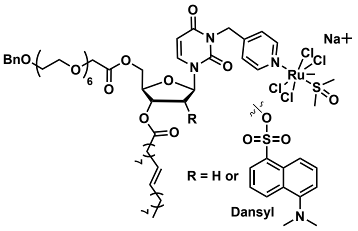	N.A.	12.1 ± 3 (MDA-MB-231)<br>12.7 ± 4 (MCF7)<br>(when R= H)<br>Concentrations correspond to the effective metal concentration (15% mol/mol) carried by nanoaggregates	Suppression of tumor growth (15 mg/kg/week, 28 days) in athymic nude mice bearing MCF7 cells. No apparent toxicity. [34]
**34**	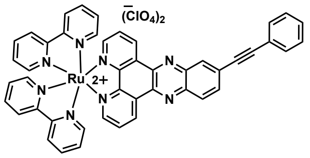	N.A.	17.2 ± 0.9 (MDA-MB-231)<br>74.9 ± 3.5 (MCF7)	Inhibition of cancer cell proliferation and metastasis (5 μM, 72 h) in a xenograft model of human MDA-MB-231 cells in zebrafish. [35]

References

1. Castonguay, A.; Doucet, C.; Juhas, M.; Maysinger, D. New Ruthenium(II)–Letrozole Complexes as Anticancer Therapeutics. *J. Med. Chem.* **2012**, *55*, 8799-8806, doi:10.1021/jm301103y.
2. Golbaghi, G.; Haghdoost, M.M.; Yancu, D.; López de los Santos, Y.; Doucet, N.; Patten, S.A.; Sanderson, J.T.; Castonguay, A. Organoruthenium(II) Complexes Bearing an Aromatase Inhibitor: Synthesis, Characterization, in Vitro Biological Activity and in Vivo Toxicity in Zebrafish Embryos. *Organometallics* **2019**, *38*, 702-711, doi:10.1021/acs.organomet.8b00897.
3. Golbaghi, G.; Pitard, I.; Lucas, M.; Haghdoost, M.M.; López de los Santos, Y.; Doucet, N.; Patten, S.A.; Sanderson, J.T.; Castonguay, A. Synthesis and biological assessment of a ruthenium(II) cyclopentadienyl complex in breast cancer cells and on the development of zebrafish embryos. *Eur. J. Med. Chem.* **2020**, doi: 10.1016/j.ejmech.2019.112030.
4. Zamora, A.; Denning, C.A.; Heidary, D.K.; Wachter, E.; Nease, L.A.; Ruiz, J.; Glazer, E.C. Ruthenium-containing P450 inhibitors for dual enzyme inhibition and DNA damage. *Dalton Trans.* **2017**, *46*, 2165-2173, doi:10.1039/C6DT04405K.
5. Pigeon, P.; Top, S.; Vessières, A.; Huché, M.; Hillard, E.A.; Salomon, E.; Jaouen, G. Selective Estrogen Receptor Modulators in the Ruthenocene Series. Synthesis and Biological Behavior. *J. Med. Chem.* **2005**, *48*, 2814-2821, doi:10.1021/jm049268h.
6. Zhao, X.; Li, M.; Sun, W.; Fan, J.; Du, J.; Peng, X. An estrogen receptor targeted ruthenium complex as a two-photon photodynamic therapy agent for breast cancer cells. *Chem. Commun.* **2018**, *54*, 7038-7041, doi:10.1039/C8CC03786H.
7. Singh, A.K.; Saxena, G.; Sahabjada; Arshad, M. Synthesis, characterization and biological evaluation of ruthenium flavanol complexes against breast cancer. *Spectrochim Acta A. Mol. Biomol. Spectrosc.* **2017**, *180*, 97-104, doi: 10.1016/j.saa.2017.02.056.
8. Roy, S.; Sil, A.; Chakraborty, T. Potentiating apoptosis and modulation of p53, Bcl2, and Bax by a novel chrysin ruthenium complex for effective chemotherapeutic efficacy against breast cancer. *J. Cell. Physiol.* **2019**, *234*, 4888-4909, doi: 10.1002/jcp.27287.
9. Schobert, R.; Seibt, S.; Effenberger-Neidnicht, K.; Underhill, C.; Biersack, B.; Hammond, G.L. (Arene)Cl2Ru(II) complexes with N-coordinated estrogen and androgen isonicotinates: Interaction with sex hormone binding globulin and anticancer activity. *Steroids* **2011**, *76*, 393-399, doi: 10.1016/j.steroids.2010.12.009.
10. Ruiz, J.; Rodríguez, V.; Cutillas, N.; Espinosa, A.; Hannon, M.J. A Potent Ruthenium(II) Antitumor Complex Bearing a Lipophilic Levonorgestrel Group. *Inorg. Chem.* **2011**, *50*, 9164-9171, doi:10.1021/ic201388n.
11. Lv, G.; Qiu, L.; Li, K.; Liu, Q.; Li, X.; Peng, Y.; Wang, S.; Lin, J. Enhancement of therapeutic effect in breast cancer with a steroid-conjugated ruthenium complex. *New J. Chem.* **2019**, *43*, 3419-3427, doi: 10.1039/C8NJ04159H.
12. Lopes, J.C.S.; Damasceno, J.L.; Oliveira, P.F.; Guedes, A.P.M.; Tavares, D.C.; Deflon, V.M.; Lopes, N.P.; Pivatto, M.; Batista, A.A.; Maia, P.I.S., et al. Ruthenium(II) Complexes Containing Anti-Inflammatory Drugs as Ligands: Synthesis, Characterization and in vitro Cytotoxicity Activities on Cancer Cell Lines. *J. Braz. Chem. Soc.* **2015**, *26*, 1838-1847, doi: 10.5935/0103-5053.20150161.
13. Mandal, P.; Kundu, B.K.; Vyas, K.; Sabu, V.; Helen, A.; Dhankhar, S.S.; Nagaraja, C.M.; Bhattacherjee, D.; Bhabak, K.P.; Mukhopadhyay, S. Ruthenium(ii) arene NSAID complexes: inhibition of cyclooxygenase and antiproliferative activity against cancer cell lines. *Dalton Trans.* **2018**, *47*, 517-527, doi: 10.1039/C7DT03637J.
14. Chatterjee, S.; Biondi, I.; Dyson, P.J.; Bhattacharyya, A. A bifunctional organometallic ruthenium drug with multiple modes of inducing apoptosis. *J. Biol. Inorg. Chem.* **2011**, *16*, 715-724, doi: 10.1007/s00775-011-0772-0.
15. Du, J.; Zhang, E.; Zhao, Y.; Zheng, W.; Zhang, Y.; Lin, Y.; Wang, Z.; Luo, Q.; Wu, K.; Wang, F. Discovery of a dual-targeting organometallic ruthenium complex with high activity inducing early stage apoptosis of cancer cells. *Metallomics* **2015**, *7*, 1573-1583, doi: 10.1039/C5MT00122F.
16. Alves Rico, S.R.; Abbasi, A.Z.; Ribeiro, G.; Ahmed, T.; Wu, X.Y.; de Oliveira Silva, D. Diruthenium(ii,iii) metallodrugs of ibuprofen and naproxen encapsulated in intravenously injectable polymer–lipid nanoparticles exhibit enhanced activity against breast and prostate cancer cells. *Nanoscale* **2017**, *9*, 10701-10714, doi: 10.1039/C7NR01582H.
17. Wang, Z.; Qian, H.; Yiu, S.-M.; Sun, J.; Zhu, G. Multi-targeted organometallic ruthenium(II)–arene anticancer complexes bearing inhibitors of poly(ADP-ribose) polymerase-1: A strategy to improve cytotoxicity. *J. Inorg. Biochem.* **2014**, *131*, 47-55, doi: 10.1016/j.jinorgbio.2013.10.017.

18. Pracharova, J.; Novohradsky, V.; Kostrhunova, H.; Štarha, P.; Trávníček, Z.; Kasparkova, J.; Brabec, V. Half-sandwich Os(ii) and Ru(ii) bathophenanthroline complexes: anticancer drug candidates with unusual potency and a cellular activity profile in highly invasive triple-negative breast cancer cells. *Dalton Transactions* **2018**, *47*, 12197-12208, doi:10.1039/C8DT02236D.
19. Naves, M.A.; Graminha, A.E.; Vegas, L.C.; Luna-Dulcey, L.; Honorato, J.; Menezes, A.C.S.; Batista, A.A.; Cominetti, M.R. Transport of the Ruthenium Complex [Ru(GA)(dppe)$_2$]PF$_6$ into Triple-Negative Breast Cancer Cells Is Facilitated by Transferrin Receptors. *Mol. Pharm.* **2019**, *16*, 1167-1183, doi: 10.1021/acs.molpharmaceut.8b01154.
20. Oliveira, K.M.; Corrêa, R.S.; Barbosa, M.I.F.; Ellena, J.; Cominetti, M.R.; Batista, A.A. Ruthenium(II)/triphenylphosphine complexes: An effective way to improve the cytotoxicity of lapachol. *Polyhedron* **2017**, *130*, 108-114, doi: 10.1016/j.poly.2017.04.005.
21. Côrte-Real, L.; Karas, B.; Gírio, P.; Moreno, A.; Avecilla, F.; Marques, F.; Buckley, B.T.; Cooper, K.R.; Doherty, C.; Falson, P., et al. Unprecedented inhibition of P-gp activity by a novel ruthenium-cyclopentadienyl compound bearing a bipyridine-biotin ligand. *Eur. J. Med. Chem.* **2019**, *163*, 853-863, doi: 10.1016/j.ejmech.2018.12.022.
22. Côrte-Real, L.; Karas, B.; Brás, A.R.; Pilon, A.; Avecilla, F.; Marques, F.; Preto, A.; Buckley, B.T.; Cooper, K.R.; Doherty, C., et al. Ruthenium–Cyclopentadienyl Bipyridine–Biotin Based Compounds: Synthesis and Biological Effect. *Inorg. Chem.* **2019**, *58*, 9135-9149, doi: 10.1021/acs.inorgchem.9b00735.
23. Morais, T.S.; Silva, T.J.L.; Marques, F.; Robalo, M.P.; Avecilla, F.; Madeira, P.J.A.; Mendes, P.J.G.; Santos, I.; Garcia, M.H. Synthesis of organometallic ruthenium(II) complexes with strong activity against several human cancer cell lines. *J. Inorg. Biochem.* **2012**, *114*, 65-74, doi: 10.1016/j.jinorgbio.2012.04.014.
24. Mendes, N.; Tortosa, F.; Valente, A.; Marques, F.; Matos, A.; Morais, T.S.; Tomaz, A.I.; Gärtner, F.; Garcia, M.H. In Vivo Performance of a Ruthenium-cyclopentadienyl Compound in an Orthotopic Triple Negative Breast Cancer Model. *Anti-Cancer Agent Me.* **2017**, *17*, 126-136, doi: 10.2174/1871520616666160922165133.
25. Frik, M.; Martínez, A.; Elie, B.T.; Gonzalo, O.; Ramírez de Mingo, D.; Sanaú, M.; Sánchez-Delgado, R.; Sadhukha, T.; Prabha, S.; Ramos, J.W., et al. In Vitro and in Vivo Evaluation of Water-Soluble Iminophosphorane Ruthenium(II) Compounds. A Potential Chemotherapeutic Agent for Triple Negative Breast Cancer. *J. Med. Chem.* **2014**, *57*, 9995-10012, doi: 10.1021/jm5012337.
26. Nhukeaw, T.; Temboot, P.; Hansongnern, K.; Ratanaphan, A. Cellular responses of BRCA1-defective and triple-negative breast cancer cells and in vitro BRCA1 interactions induced by metallo-intercalator ruthenium(II) complexes containing chloro-substituted phenylazopyridine. *BMC Cancer* **2014**, *14*, 73-73, doi: 10.1186/1471-2407-14-73.
27. Cao, W.; Zheng, W.; Chen, T. Ruthenium polypyridyl complex inhibits growth and metastasis of breast cancer cells by suppressing FAK signaling with enhancement of TRAIL-induced apoptosis. *Sci. Rep.* **2015**, *5*, 9157-9157, doi: 10.1038/srep09157.
28. Montani, M.; Pazmay, G.V.B.; Hysi, A.; Lupidi, G.; Pettinari, R.; Gambini, V.; Tilio, M.; Marchetti, F.; Pettinari, C.; Ferraro, S., et al. The water soluble ruthenium(II) organometallic compound [Ru(p-cymene)(bis(3,5 dimethylpyrazol-1-yl)methane)Cl]Cl suppresses triple negative breast cancer growth by inhibiting tumor infiltration of regulatory T cells. *Pharmacol. Res.* **2016**, *107*, 282-290, doi: 10.1016/j.phrs.2016.03.032.
29. Shen, J.; Kim, H.-C.; Wolfram, J.; Mu, C.; Zhang, W.; Liu, H.; Xie, Y.; Mai, J.; Zhang, H.; Li, Z., et al. A Liposome Encapsulated Ruthenium Polypyridine Complex as a Theranostic Platform for Triple-Negative Breast Cancer. *Nano Lett.* **2017**, *17*, 2913-2920, doi: 10.1021/acs.nanolett.7b00132.
30. Popolin, C.P.; Reis, J.P.B.; Becceneri, A.B.; Graminha, A.E.; Almeida, M.A.P.; Corrêa, R.S.; Colina-Vegas, L.A.; Ellena, J.; Batista, A.A.; Cominetti, M.R. Cytotoxicity and anti-tumor effects of new ruthenium complexes on triple negative breast cancer cells. *PLoS One* **2017**, *12*, e0183275, doi: 10.1371/journal.pone.0183275.
31. Becceneri, A.B.; Popolin, C.P.; Plutin, A.M.; Maistro, E.L.; Castellano, E.E.; Batista, A.A.; Cominetti, M.R. The trans-[Ru(PPh$_3$)$_2$(N,N-dimethyl-N′-thiophenylthioureato-k$_2$O,S)(bipy)]PF$_6$ complex has pro-apoptotic effects on triple negative breast cancer cells and presents low toxicity in vivo. *J. Inorg. Biochem.* **2018**, *186*, 70-84, doi: 10.1016/j.jinorgbio.2018.05.011.
32. Colina-Vegas, L.; Oliveira, K.M.; Cunha, B.N.; Cominetti, M.R.; Navarro, M.; Azevedo Batista, A. Anti-Proliferative and Anti-Migration Activity of Arene–Ruthenium(II) Complexes with Azole Therapeutic Agents. *Inorganics* **2018**, *6*, 132, doi: 10.3390/inorganics6040132.

33. Silva, H.V.R.; Dias, J.S.M.; Ferreira-Silva, G.Á.; Vegas, L.C.; Ionta, M.; Corrêa, C.C.; Batista, A.A.; Barbosa, M.I.F.; Doriguetto, A.C. Phosphine/diimine ruthenium complexes with Cl^-, CO, NO^+, NO_2^-, NO_3^- and pyridine ligands: Pro-apoptotic activity on triple-negative breast cancer cells and DNA/HSA interactions. *Polyhedron* **2018**, *144*, 55-65, doi: 10.1016/j.poly.2018.01.005.
34. Piccolo, M.; Misso, G.; Ferraro, M.G.; Riccardi, C.; Capuozzo, A.; Zarone, M.R.; Maione, F.; Trifuoggi, M.; Stiuso, P.; D'Errico, G., et al. Exploring cellular uptake, accumulation and mechanism of action of a cationic Ru-based nanosystem in human preclinical models of breast cancer. *Sci. Rep.* **2019**, *9*, 7006, doi: 10.1038/s41598-019-43411-3.
35. Zhao, X.; Li, L.; Yu, G.; Zhang, S.; Li, Y.; Wu, Q.; Huang, X.; Mei, W. Nucleus-enriched Ruthenium Polypyridine Complex Acts as a Potent Inhibitor to Suppress Triple-negative Breast Cancer Metastasis In vivo. *Comput. Struct. Biotechnol. J.* **2019**, *17*, 21-30, doi: 10.1016/j.csbj.2018.11.010.
